# Characterization of the *Arion vulgaris* pedal gland system

**DOI:** 10.1002/jmor.21231

**Published:** 2020-07-10

**Authors:** Sophie Greistorfer, Johannes Suppan, Norbert Cyran, Waltraud Klepal, Robert Farkas, Livia Rudoll, Janek von Byern

**Affiliations:** ^1^ Faculty of Life Science, Core Facility Cell Imaging and Ultrastructure Research University of Vienna Vienna Austria; ^2^ Department of Orthopaedic and Trauma Surgery Medical University of Vienna Vienna Austria; ^3^ Laboratory of Developmental Genetics Institute of Experimental Endocrinology, Biomedical Research Center, Slovak Academy of Sciences Bratislava Slovakia; ^4^ Faculty of Life Science, Department of Integrative Zoology University of Vienna Vienna Austria; ^5^ Austrian Cluster for Tissue Regeneration Ludwig Boltzmann Institute for Experimental and Clinical Traumatology Vienna Austria

**Keywords:** anatomy, Gastropoda, histochemistry, mantle epithelium, mucus

## Abstract

The most common European gastropod species, *Arion vulgaris*, is one of the most troublesome pests for private garden owners and commercial agriculturists. The sticky and hard to remove secretion produced by these animals allows them to overcome most artificial and natural barriers. However, this highly adherent biopolymer has recently shown great potential for novel wound‐healing applications in medicine. Nevertheless, our knowledge of the underlying gland system is still limited and few studies on the ventral gland system are available. We studied the lateral and ventral pedal glands in *Arion vulgaris* to determine their secretory content histochemically and through lectin assays. Using these histological and histochemical methods we differentiate five gland types with different mucus composition in the lateral pedal region of the foot of *Arion vulgaris*. These contain sulphated and carboxylated mucosubstances (positive Alcian blue staining) but lack hexose‐containing mucosubstances (negative PAS staining). In the ventral pedal region, four gland types can be differentiated producing sulphated and carboxylated mucosubstances. Within the ventral mucus, a high affinity for the lectins PNA and WGA is observed. While the lateral glands are histochemically negative for PAS, a positive staining with the lectin JAC is observed. *Arion vulgaris* shows clear morphological differences from other arionid species. This raises the question whether the variation in the chemistry of the secretory material and mucus composition is the result of different functions and/or is related to the animals' different environmental conditions. A comparison of some glands of *Arion vulgaris* with those of the helicid species *Helix pomatia* and *Cepaea hortensis* indicates morphological similarities.

## Graphical abstract

- The microanatomy of the *Arion vulgaris* pedal gland system is described in detail.
- Histochemically, the dorsal glands mostly consists of acidic proteins while ventrally also sugar as α‐L‐fucose could be detected.
- The data given for *Arion vulgaris* gland system show similarities to the gland morphologies of helicid species like *Helix pomatia* and *Cepaea hortensis* but differ clearly to those in other slugs.

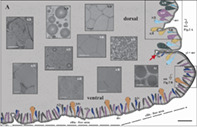

## 1 INTRODUCTION

Gastropods occur in a variety of habitats (terrestrial and fresh and saltwater environments) and can produce considerable amounts of mucus, for example, for defence, adhesion, locomotion or lubrication (Smith, 2006, 2010). Furthermore, gastropod secretions are promising for medical (Li et al., 2017; Tsoutsos, Kakagia, & Tamparopoulos, 2009), cosmetic (Pons, Koenig, Michelot, Mayer, & Frossard, 1998), and/or industrial applications (Davies & Hawkins, 1998; Shirtcliffe, McHale, & Newton, 2012) and are known to have been used since ancient times (Meyer‐Rochow, 2017).

Although gastropod mucus appears to offer a range of industrial applications, surprisingly little is known about the mucus‐producing glands in this group of molluscs. In an earlier study on the pedal gland system in the helicid species *Helix pomatia* and *Cepaea hortensis*, we reported differences concerning gland number, size, and content (Greistorfer et al., 2017; von Byern et al., 2018). While in *Helix* three different glands were observed in the dorsal epithelium (Greistorfer et al., 2017), in *Cepaea* four glands could be distinguished in the same location (von Byern et al., 2018). The glands also differ histochemically between the two species: Compared to *Cepaea*, *Helix* glands contain different sugar moieties and lack basic proteins. In both species, two ventrally located gland types were described that are positive for acidic glycoproteins only, but with different sugar moieties (Greistorfer et al., 2017; von Byern et al., 2018).

With this study, we extend our investigation of the gastropod pedal gland and aim to characterise Arionidae. These gastropod group lack the protective shell of helicid species and therefore secrete an extremely stiff mucus when threatened by predators (Mair & Port, 2002; Martin & Deyrup‐Olsen, 1986). However, large differences in their mucus‐producing glands are known to exist both between and within species: In *Arion ater*, two (Barr, 1927) to three (Wondrak, 1967) types of glands have been described in the ventral and lateral epithelia. In the related species *Arion rufus*, four ventral glands have been found (Chétail & Binot, 1967), but these authors did not provide any details on the lateral subepithelial glands in their *Arion* species. More recently, Wondrak (2012) revealed four different epithelial regions in *Arion rufus*, each showing reactivity to specific lectins and involvement in mucus formation:

1. A protein gland region, located in the anterior region of the foot showing a strong positive reaction to PAS and the lectins PNA (peanut agglutinin), RCA (*Ricinus communis* agglutinin), and HPA (*Helix pomatia* agglutinin). However, it is negative for Alcian blue 4.0 and Safranin O. This gland is probably responsible for turning the trail mucus (used for locomotion) into the strong glue‐mucus (used for attachment).
2. A suprapedal gland region, in which acidic mucosubstances (Alcian blue 4.0 staining) show a weak reaction to the lectin GSL‐1 (*Griffonia simplicifolia* lectin I) as well as a weak reaction to the lectins RCA and HPA. These might contribute to the trail mucus, similar to the protein gland.
3. A ventral surface region of the head, which is PAS positive but reacts neither to Alcian blue nor to Safranin O. Most of the cells bind strongly to HPA and RCA and to a lower extent to GSL‐I and WGA (wheat germ agglutinin).
4. An anterior part of the ventral mantle, reactive to Alcian blue, while Safranin O stains only part of the granules. This gland reacts to none of the applied lectins. According to Wondrak (2012), the latter two gland regions might be involved in the production of the lateral mucus, although they are ventrally located.

Although previous examinations of the pedal gland system in the Arionidae provide a good overview of the number of ventral glands, the gland system of *Arion vulgaris* has only been marginally examined. The aim of the current study of the lateral and ventral pedal glands in *Arion vulgaris* is to determine their secretory content histochemically and by lectin affinity tests. A comparison with earlier studies on *Helix pomatia* and *Cepaea hortensis* (Greistorfer et al., 2017; von Byern et al., 2018) is expected to show structural and chemical differences in the gland systems and mucus of these three common European terrestrial gastropods.

## 2 MATERIAL AND METHODS

Adult *Arion vulgaris* Moquin‐Tandon, 1855 (*n* = 6) specimens were collected in Lower Austria (GPS data: N48°4′55.35″; E15°28′50.34″) and subsequently fixed for detailed microanatomical and histochemical characterization, following the methods described in Greistorfer et al. (2017). DNA‐based species identification (barcoding data) of two *Arion vulgaris* samples are available in the BOLD systems database (https://www.boldsystems.org/) as AMOL575‐19 and AMOL576‐19, uploaded by the National History Museum of Vienna, Austria.

### 2.1 Morphology

For the ultrastructural characterization, two samples were immersed in 2.5% glutaraldehyde buffered with sodium cacodylate (0.1 mol l^−1^, pH 7.4, plus 10% sucrose) for 5 hr at room temperature. Afterwards, the samples were washed in the same buffer, post‐fixed for 1 hr in 1% osmium tetroxide (again in 0.1 mol l^−1^ sodium cacodylate buffer at pH 7.4), stepwise dehydrated, and finally embedded in Epon epoxy resin (AGAR 100, Co. Agar Scientific Ltd, United Kingdom). Polymerisation took place at 60°C for 3 days.

Semithin sections (1 μm thickness) of the resin samples were cut with a Leica UC7 ultramicrotome (Co. Leica Microsystems GmbH, Germany) and stained with Toluidine blue. Ultrathin sections (70 nm thick) were made with an ultra‐diamond knife (Co. Diatome AG, Switzerland) on a Leica UC7 ultramicrotome, mounted on copper grids, stained with Richardson solution, and visualised with a Zeiss Libra 120 transmission electron microscope (TEM) (Co. Carl Zeiss AG, Germany) at 120 kV.

For scanning electron microscopy (SEM), two animals were frozen in liquid nitrogen, freeze‐dried (Mod. LyovacGT2, Co. Leybold‐Heraeus GmbH, Germany), coated with gold in a sputter coater (Mod. 108, Co. Agar Scientific Ltd, UK), and observed with a scanning electron microscope JEOL IT 300 at 15 kV. For histochemical characterization two samples were fixated and proceeded as explained in Greistorfer et al. (2017).

To visualise the glands in the dorsal and ventral epithelium a schematic drawing was made using Illustrator CS6 (Adobe Systems, San Jose). All measurements, including the gland dimension, were made with Photopshop CS6 (Adobe Systems, San Jose). The gland length was measured on semithin sections (1 μm), the granules within the glands and the dimensions/height of the epithelia were measured on ultrathin sections (70 nm thick). Every gland was measured from the opening of the duct to the bottom of the gland. Only glands with complete extension were taken. The granules were measured from top to bottom and from right to left. For every final data, six measurements were taken and then the mean value was calculated.

To give a good overview and compare the glands to each other these measurements of the glands/granules were made. A detailed statistic evaluation is missing, because the gland size in different body regions varied a lot and it was not possible to get sufficient measurements. To keep the measurement inaccuracy low, only glands with an open duct were measured.

### 2.2 Mucus chemistry

For the chemical analyses of the pedal glands, samples from all regions of the foot (anterior, mid‐region and posterior) of two animals were fixed in Carnoy's solution (Kiernan, 1999) for 3 hr at 25°C and then sectioned into 1 mm slices with a vibratome (Leica VT 1200S, Co. Leica Biosystems GmbH, Germany). Afterwards, the samples were cleared in methylbenzoate, transferred to benzene, and infiltrated overnight with paraffin. Sections (5–7 μm thick) were cut with a rotary microtome (Leica RM2265, Co. Leica Biosystems GmbH, Germany), mounted on glass slides with Ruyter fluid (Ruyter, 1931) and dried at room temperature before use. Samples of the ventral mucus were collected by having four individual species crawl over glass slides until it was fully covered. The lateral mucus samples were collected by attaching the glass slides to the animals’ bodies, again until it was fully covered. All collected mucus samples were air‐dried overnight before staining. Following the methodological description of Greistorfer et al. (2017), histochemical analyses included the detection of hexose‐containing mucosubstances by periodic acid Schiff (PAS) staining. Further determination of the sugar moieties was performed with fluorescence‐labelled lectin affinity tests using UEA II, PNA, SBA, WGA, GNA from Co. EY Laboratories and all other 24 lectins from Co. Vector Laboratories. Further details of the tested lectins and their sugar affinities (following the nomenclature by Van Damme, Peumans, Pustai, & Bardocz, 1998) given for the ventral mucus as well as the glands can be found in Table 1. Labelling took place on isolated mucus samples as well as paraffin‐embedded samples, following the protocol of Greistorfer et al. (2017).

**TABLE 1:** Summary of the different lectins tested on the trail mucus as well as the lateral and ventral glands of *Arion vulgaris*

Lectin type	Abbrevation	Specifity	Ventral mucus	Lateral mucus	Lateral glands	Ventral glands
Concanavalin agglutinin	ConA	α‐linked mannose	+	−	−	−
*Datura stramonium* agglutinin	DSA	N‐acetylglucosamine (GlcNAc) (oligomers)	−	−	−	−
*Erythrina cristagalli* lectin	ECL	N‐Acetyllactoseamine>N‐Acetylgalactosamine>Galactose	−	−	−	−
*Euonymus europaeus* agglutinin	EEA	Galactose‐linked (gala[1,3]gal)	–	−	−	−
*Galanthus nivalis* agglutinin	GNA	Mannose‐linked (Mana[1,3]man)	**+**	−	–	−
Biotinylated *Griffonia* (Bandeiraea) *simplicifolia* lectin	GSL II	N‐Acetylgalactosamine (oligomer>monomer)	−	−	–	−
*Artocarpus integrifolia* lectin	JACALIN	Galactose‐linked to N‐acetylgalactosamine type (Galβ[1,3]GalNAc)	−	**++**	**++**	−
*Maackia amurensis* lectin	MAL	N‐acetylneuraminic acid linked to Galactose and N‐Acetylglucosamine (Neu5Ac(α2–6)gal(β1–4)GlcNAc)	−	−	−	−
Peanut agglutinin	PNA	Galactose.Linked to N‐acetylgalactosamine (Galβ[1,3]GalNAc) > GalNH_2_ > lac	**++**	−	+ **−**	−
*Sambucus nigra* agglutinin	SNA	Sialic acid‐linked to N‐acetylgalactosamine or galactose	−	−	−	−
*Datura stramonium* lectin	STL	N‐acetyl‐D‐glucosamin type (GlcNAcβ[1,4])	−	−	−	**+**
*Vicia villosa* lectin	VVL	N‐acetylgalactosamine type (GalNAca[1,3]gal) > blood goup A	**+**	**+**	**+**	**+ −**
Wheat germ agglutinin	WGA	N‐acetylglucosamine (oligomer>monomer>NANA)	**++**	−	**+**	−
*Dolichos biflorus* agglutinin	DBA	N‐acetylgalactosamine (GalNAc)	−	−	−	−
Biotinylated *Griffonia* (Bandeiraea) *simplicifolia* lectin I	GSL I	N‐acetylgalactosamine (GalNAc) > gal	−	−	−	−
*Lens culinaris* agglutinin	LCA	Mannose>glucose	−	−	−	−
*Phaseolus vulgaris* lectin	PHA‐E	Galactose‐linked to N‐acetylglucosamine and mannose (Galb(1,4)GlcNAcb(1,2)man))	−	−	−	−
*Phaseolus vulgaris* lectin L	PHA‐L	Galactose‐linked to N‐Acetylglucosamine and mannose (gal (1,4)GlcNAc(1,2)man)	−	−	−	−
*Pisum sativum* agglutinin	PSA	Mannose>glucose	−	−	−	−
Soybean agglutinins	SBA	N‐Acetylgalactosamine (GalNAc) > gal	−	−	−	−
*Styphnolobium japonicum* agglutinin	SJA	N‐Acetylgalactosamine (GalNAc) > gal	−	−	−	−
*Ulex europaeus* agglutinin	UEA I	α‐Linked fucose>Fuca(1‐2)Galβ(1‐3)GalNA	−	−	−	−
*Ulex europaeus* agglutinin	UEA II	N‐acetylglucosamine (oligomers) > Fuca(1‐2)Galβ(1‐3)GalNA	**++**	−	−	**++**
Succinylated wheat germ agglutinin	WGAs	N‐acetylglucosamine, no sialic acid residues	−	**+**	**+**	−

Sulphated and carboxylated acidic mucosubstances were stained at pH 1.0 and 2.5 using Alcian blue G8X staining (McManus & Mowry, 1960), at pH 4.3 using Toluidine blue O (Mulisch & Welsch, 2010), and at pH 6, 8, 9.5, and 10.5 for basic proteins using Biebrich scarlet staining (Kiernan, 1999; Spicer & Lillie, 1961). A combination of Alcian blue and PAS staining was used to verify the presence of acidic glycoproteins, while Safranin O staining (Böhm & Oppel, 1919) was applied to confirm *polyanionic* proteoglycans in the glands.

Calcium was determined by Alizarin red S (Kiernan, 1999) and von Kossa staining (Sheehan & Hrapchack, 1980). To document the presence of L‐3,4‐dihydroxyphenylalanine (L‐DOPA) in tissue and isolated mucus samples, the samples were stained according to the protocol by Arnow (1937). Samples from the tube‐dwelling polychaete *Sabellaria alveolata* were used as a positive control (Becker, Lambert, Lejeune, Lanterbecq, & Flammang, 2012). Lipids were visualized with Sudan black B (Mulisch, & Welsch, 2010) on isolated ventral and lateral mucus samples only.

### 2.3 Mucus element analysis

For the histochemical analyses, fresh lateral and ventral mucus from four individuals was collected on standard SEM stubs, consisting of aluminium and copper (Co. Gröpl, Austria) until the whole stub was covered. All samples were then air‐dried and analysed using energy dispersive x‐ray spectroscopy (EDX) with the x‐ray microanalysis software (Software Team, Version 4.3, Co. Ametek, Germany) in the SEM JEOL IT 300 at 20 kV. The collecting time for the elements in the mucus was set to 30 s for the selected areas and 4 hr for dot mapping, both with a ~ 30% dead time. At least 5–7 point measurements from each mucus type (lateral and ventral) and each of the four individuals were taken, and these are summarised in Table 2 as a range between minimum and maximum values. For the comparison of the different mucus types, atom % (at.%) was used, values below 0.1 at.% are ignored due to the detection limit of this semiquantitative method.

**TABLE 2:** Morphology and chemical comparison of epithelial glands between *Arion vulgaris* and the helicid species *Cepaea hortensis* (von Byern et al., 2018) and *Helix pomatia* (Greistorfer et al., 2017) [Color table can be viewed at wileyonlinelibrary.com]

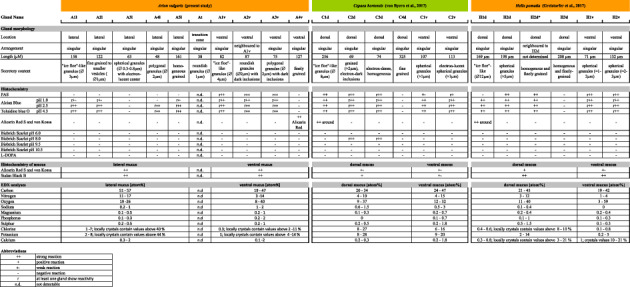

### 2.4 Gland terminology and imaging

According to the gastropod gland nomenclature of Smith (2006), the gland cells were named as follows: The first letter of the genus name *Arion*, a sequential number, and the acronym “l” for lateral or “v” for ventral.

**FIGURE 1:**
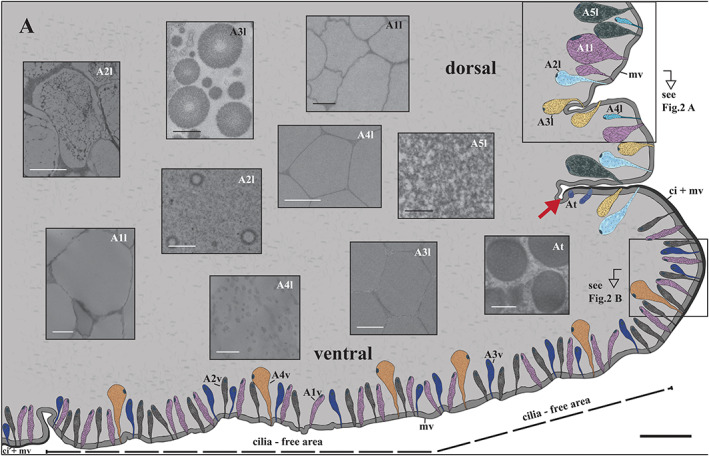
*Arion vulgaris*, schematic overview of the pedal gland system. The lateral epithelium is covered by a microvilli layer (mv), while ventrally a ciliary border (ci) is additionally present in the periphery and centre of the sole. Laterally, five gland types (A1l, A2l, A3l, A4l, and A5l) are present, which differ in their secretory content (see inserts). In the ventral region, the gland types A1v, A2v, and A3v are frequently present, while A4v occurs mainly in the cilia‐free periphery of the sole. The gland type At only occurs in the peripheral groove (marked by a red arrow), the transition region between the lateral and ventral epithelium. Scale bar in main image = 250 μm, in the inserts A2l, A4l, A1v, A2v = 1 μm, and in the inserts A1l, A3l, A5l, A3v, A4v and At = 0.5 μm

**FIGURE 2:**
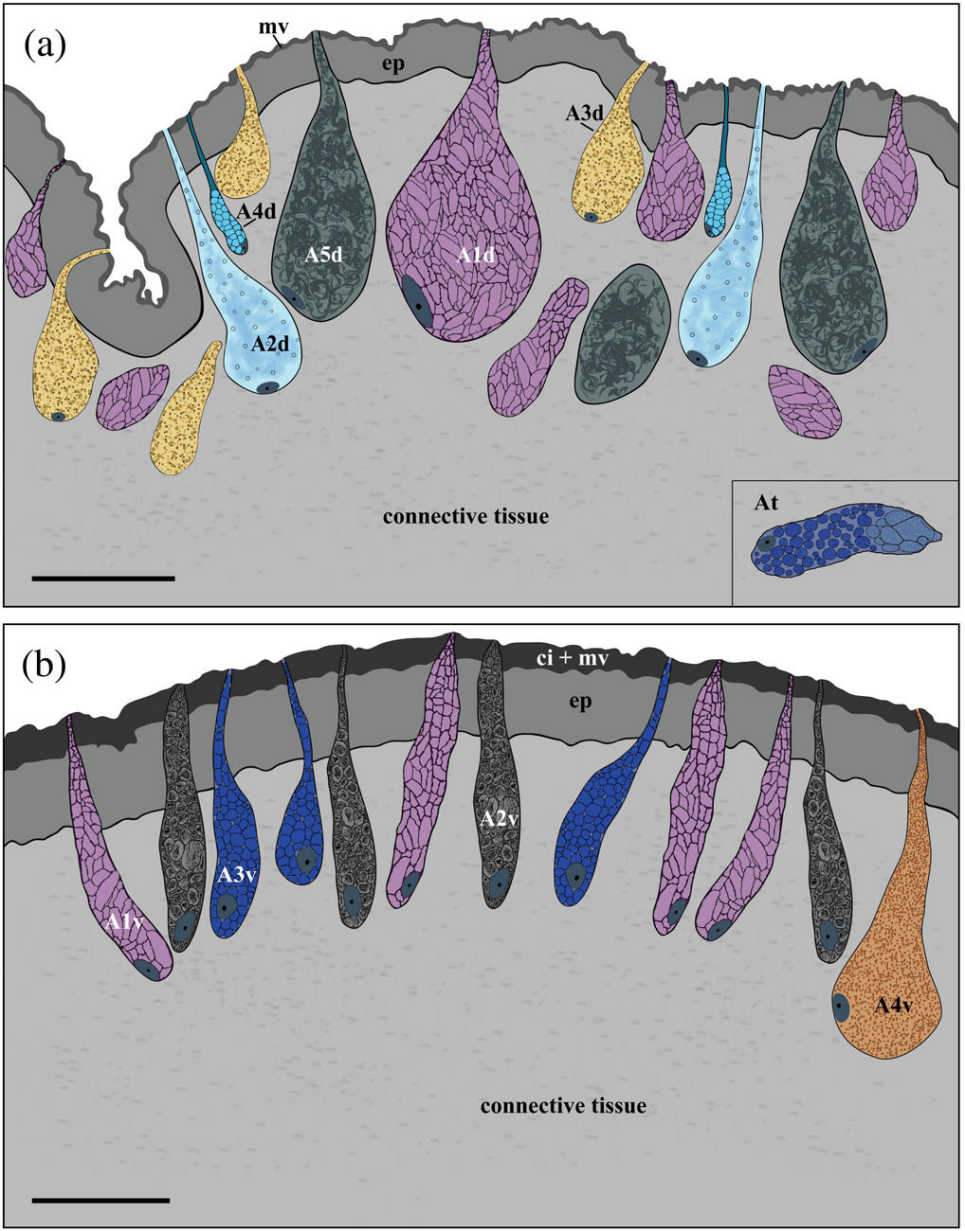
*Arion vulgaris*, subepidermal gland types in the lateral and ventral pedal integument. Detailed overview of the (a) five lateral gland types (A1l–A5l), the transitory gland type At, and (b) the four ventral gland types (A1v–A4v) and their secretory content. The lateral pedal epithelium (ep) is covered by microvilli (mv), while on the ventral side the epidermis cells have not only microvilli but also cilia (ci + mv) on their apical pole. Scale bars in a and b = 50 μm

As done earlier for *Helix pomatia* (Greistorfer et al., 2017) and *Cepaea hortensis* (von Byern et al., 2018), the schematic drawings of the glands in the lateral and ventral *Arion* epithelia (Figures 1, and 2) were based on histochemical sections. Details on the epithelial and gland dimensions as well as the granular appearance were illustrated based on the semithin and ultrathin sections. The software Illustrator CS6 (Co. Adobe Systems) was used to produce the drawings.

## 3 RESULTS

The lateral epithelial layer reaches a thickness of up to 16 μm, while the ventral layer is around 22 μm high (Figure 1); both are separated by a large groove, the transition zone. A layer of microvilli (≈0.5 μm high) is located on the lateral epithelium as well as in the peripheral zone of the ventral region. Besides the microvilli, cilia (length ≈11 μm) are also present in the transition zone and the central part of the sole. This sole centre is the part of the arionid foot which clings to various types of slippery substrate.

All gland types of the foot of *Arion vulgaris* are unicellular, subepithelial, and embedded in connective tissue. Their nucleus is situated laterally or centrally in the basal area of the gland. Laterally, five different gland types (A1l, A2l, A3l, A4l, and A5l) can be differentiated by their appearance (Figure 1), size, and secretory content, while ventrally four different glands (named A1v, A2v, A3v, and A4v) are observed. In the transition region, one gland type (At) is present. Throughout the lateral epithelium and the subepithelium, yellow‐brownish pigments can be observed, but these are absent ventrally.

### 3.1 Lateral gland morphology

**FIGURE 3:**
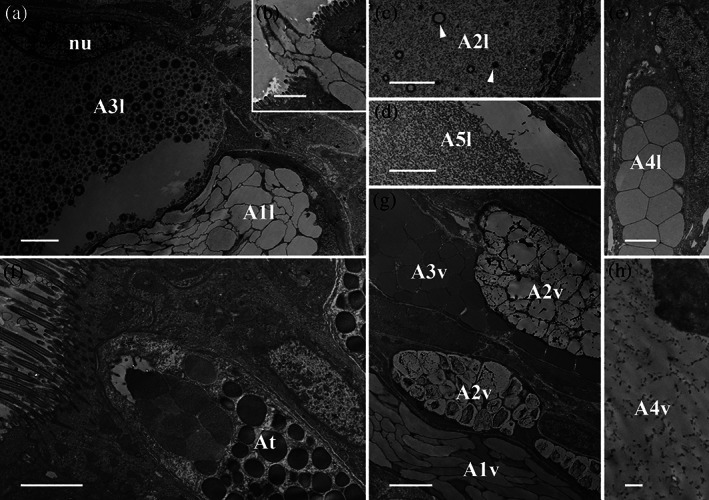
*Arion vulgaris*, transmission electron micrographs of the lateral and ventral gland types. (a) The secretory cells of A1l and A3l could be clearly differentiated in an electron microscope; A1l bears electron‐translucent “ice floes” while the granules of A3l are spherical with several concentric rings and an electron‐translucent centre. The nucleus (nu) could be seen close to the secretory content of A3l. (b) The secretory content of A1l is extruded as a whole package. (c) The content of A2l consists of granulated material of different sizes (white arrowheads) and (d) that of A5l is homogeneous granular material. (e) Gland type A4l contains polygonal granules of homogenous material. (f) Gland type At, which occurs in the transition zone between the lateral and ventral sides, contains roundish granules merging near the apical pole. (g) In the ventral region, three different gland types (A1v, A2v, and A3v) can be observed, all appearing in high abundance and containing granules, different in size and appearance. (h) The secretory content of gland type A4v is finely grained with dark grained inclusions. Scale bars in a, c, d, f, and g = 2.5 μm, in image b and e = 2 μm and in image h = 0.5 μm

**FIGURE 4:**
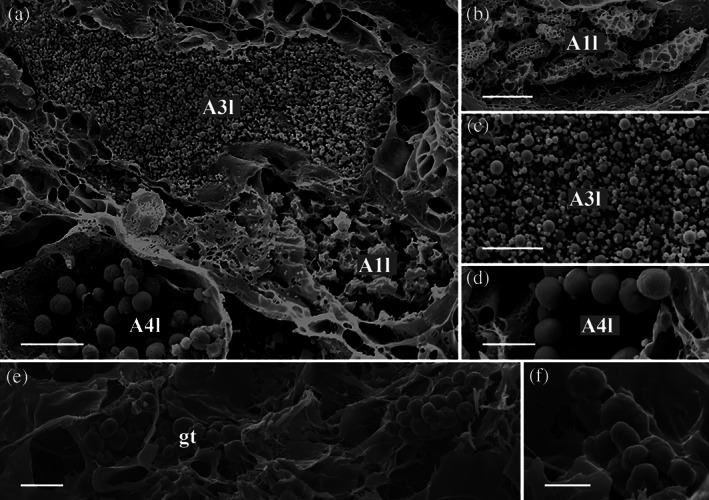
*Arion vulgaris*, scanning electron micrographs of the lateral gland types. In the freeze‐dried tissue samples, (a) three gland types could be observed, which correlate in view of its granular content with the gland types A1l, A3l, and A4l (see Figure 3 for comparison). (b) The granules of gland type A1l are oval and appear sponge‐like. (c) Gland type A3l contains granules of different sizes, while (d) gland type A4l contains roundish, evenly sized granules. Scale bar in image a = 10 μm, in image b to c = 5 μm

**A1l** is a common gland type, frequently present in the lateral area of *Arion vulgaris*, extending to a depth of around 158 μm. This gland type has a drop‐like shape (Figure 2a); its secretory material is densely packed and in the TEM (Figure 3a) it appears as electron‐translucent “ice floes” (size of ≈3 μm in the longest dimension) or as sponge‐like granules in the freeze‐dried state in the SEM (Figure 4a,b). The data indicate that the secretory material is continuously secreted (Figure 3b) and is not pinched off or temporally stored in an apical depot.

Gland type **A2l** is about 122 μm long, is also frequent in the lateral body wall, and is similar in size and shape to A1l (Figure 2a). This gland type contains fine granular material (Figure 3c) and vesicles of different sizes (from ≈0.1 μm to ≈0.6 μm) with an electron‐translucent content (Figure 3c).

Gland type **A3l** is likewise frequently present in the *Arion* lateral region but is smaller in size (measured up to 63 μm long) (Figure 2a). The gland synthesises spherical granules (with diameters ranging from ≈0.3 to ≈0.8 μm) (Figure 4a,c) with an electron‐translucent centre surrounded by several concentric rings (Figure 3a). In this study, the glands always appear filled with granules, but not as densely as the A1l or A2l glands.

Only a few glands of type **A4l** are located in the lateral region and they are shorter in length (around 48 μm) (Figure 2a) than the others. The electron‐translucent glandular material is contained in nearly spherical granules of an almost uniform size (≈2 μm) (Figure 4a,d), which appear polygonal when tightly packed (Figure 3e).

Similar to A1l, the **A5l** glands also appear large in size (approximate length ≈161 μm) and are commonly distributed, but not as frequently as A2l (Figure 2a). These glands are filled with fine and homogeneous granular material (Figure 3d) which does not appear as tightly packed as the granular material of A1l, A3l, and A4l.

The gland type **At** is only observed in the transition region between the lateral and ventral areas of the body surface (Figure 1). With a length of ≈38 μm, it is the smallest gland type found in the *Arion vulgaris* pedal system and contains roundish granules of ~1 μm in diameter, which aggregate near the apical pole (Figure 3f).

### 3.2 Lateral gland histochemistry

**FIGURE 5:**
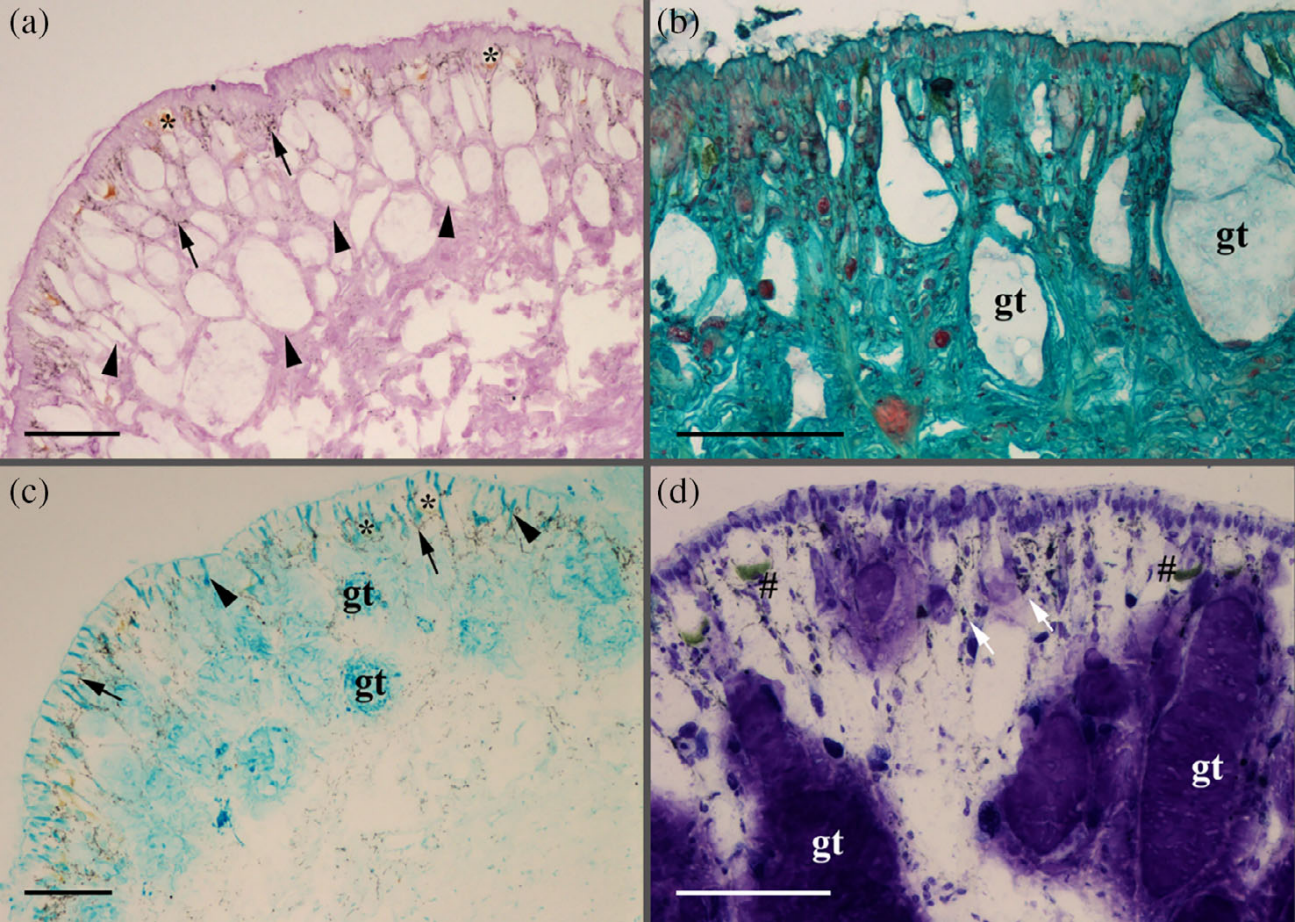
*Arion vulgaris*, histochemistry of the lateral integument. (a) Periodic acid‐Schiff reaction (PAS) confirms the presence of sugar in the connective tissue (black arrowheads) but not within the lateral glands (e.g., gland type A3l, marked by a black asterisk). The black arrows mark the yellow‐brownish pigmentation. (b) With Safranin O staining, the larger gland type (gt, which refers to a non‐identified gland type as a correlation with one of the five potential gland types failed) shows no reactivity to glycosylated proteins (mucus), as given by the bluish‐green background staining. (c) With Alcian blue staining (here shown for the pH level 1.0), some of the larger gland type (gt) and its duct (black arrowhead) react for acidic proteins. The black arrows mark the pigments within the lateral region, and the black asterisks point out gland type A3l, which show no reactivity to acidic mucosubstance staining. (d) With Toluidine blue at pH 4.3, the contents of large (gt) and small gland cells (white arrows) are strongly stained. Furthermore, a gamma metachromasia (green staining, black #) is visible in some smaller subepithelial glands. Scale bars in images a to d = 100 μm

It is difficult to clearly determine the chemical content of the five lateral gland types, mainly because the glandular material is often sparse and can only be differentiated at high resolutions. Generally, none of the five lateral glands shows a positive PAS staining, indicating that theses glands do not contain hexose‐containing mucosubstances (Figure 5a,b, Table 2); only the connective tissue around some of the glands react to the PAS staining. Nevertheless, the glands show a clear strong affinity to the lectin JAC, indicating the presence of galactose‐linked sugar moieties. To a lesser extent, the lectins VVL, WGA, and WGAs (specific for N‐acetylglucosamine types) (Table 1) are also reactive in the gland, whereas reactivity for WGA was negative in the mucus. PNA positive affinity could be observed in the glands but not mucus. Basic proteins (Biebrich scarlet staining at any pH) and L‐DOPA (Arnow staining) are absent within the glands and in the isolated mucus.

The secretory material of the large gland types (which, based on their sizes, could be A1l, A2l, and/or A5l) shows a positive Alcian blue staining at pH 1.0 (sulphated mucosubstances) (Figure 5c). Furthermore, some of the subepidermal gland types show an even stronger positive reaction to Alcian blue at pH 2.5 (data not shown), also indicating the presence of carboxylated mucosubstances. In addition, a gamma metachromasia to toluidine blue staining at pH 4.3 (Figure 5d) could be observed. The secreted mucus, collected from the lateral region, is positive for calcium (Alizarin red S and the von Kossa method) and lipids (Sudan black) (data not shown). Gland type At is too small for a light microscopic evaluation.

### 3.3 EDX‐analyses of the lateral mucus

The EDX measurements indicate the presence of carbon (51–57  at.%), nitrogen (11–17 at.%), and oxygen (19–26 at.%) in high amounts in the lateral mucus (Table 2). For the elements chlorine (1–7 at.%, locally crystals contain values above 40 at.%) and potassium (2–8 at.%, locally crystals contain values above 44 at.%), different range values could be measured as these elements are unevenly distributed in the sample (Figure S1a–c). Sodium, magnesium, phosphorus, sulphur, and calcium are also present in the mucus, however, at very low concentrations.

### 3.4 Ventral gland morphology

Gland type **A1v** (≈82 μm long) is columnar‐shaped with a narrow duct towards the epithelium surface (Figure 2b). Its granules appear irregular in size (length up to 4 μm) and are stratified like “ice‐floes”; the content is homogeneous and enclosed by an electron‐dense membrane (Figure 3g).

Gland type **A2v** is of similar shape and length (≈87 μm) as A1v and neighbors it (Figure 2b). However, the granular material of A2v clearly differs by having roundish, electron‐translucent granules (≈2 μm) with densely scattered inclusions (Figure 3g).

Gland type **A3v** is tubular to goblet‐shaped with a length of ≈75 μm (Figure 2b). Its granules are of the same diameter (≈2 μm) as those of A2v, but they are polygonal in shape and tightly packed within the gland. The content is moderately electron‐dense but lacks darker inclusions (Figure 3g).

Gland type **A4v** can only be observed in the cilia‐free peripheral region (Figure 2b). It is the longest gland (≈127 μm) in the ventral region and appears pear‐shaped. Its secretory material is finely granulated, but not as homogeneous or dense as the content of A2l and A5l (Figure 3h).

### 3.5 Ventral gland chemistry

**FIGURE 6:**
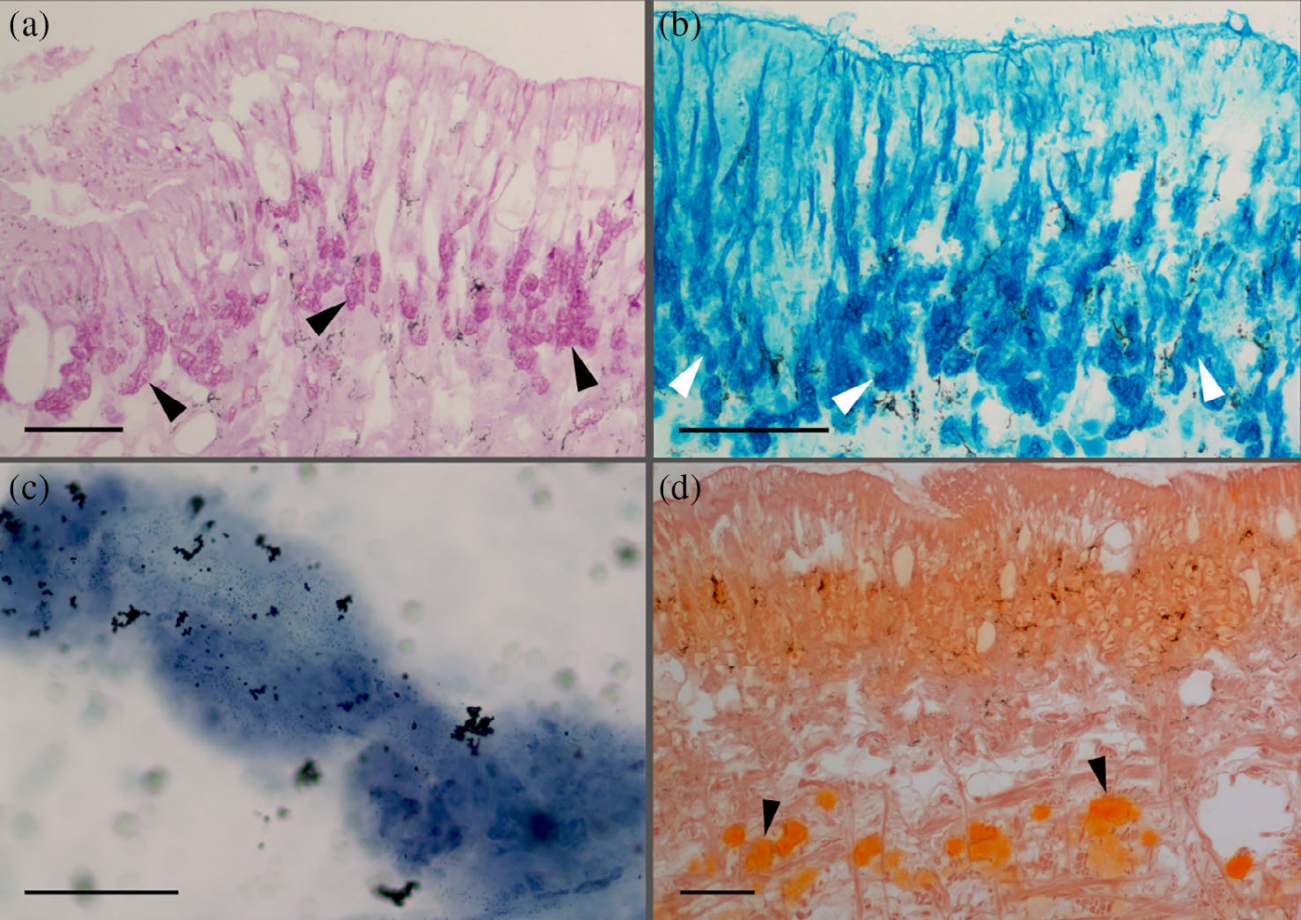
*Arion vulgaris*, histochemistry of the ventral pedal integument. (a) Strong positive periodic acid‐Schiff reaction (PAS) of the ventral gland types, as indicated by the dark pink staining (black arrowheads). (b) With Alcian blue staining (pH 2.5), a positive staining (dark blue) of the gland types is noted (white arrowheads). (c) Lipids (Sudan black B staining) could be confirmed (blue staining) in the isolated ventral mucus, while (d) Alizarin red S staining showing the presence of calcium (black arrowhead) in the subepithelial layer on the ventral side of the animal . Scale bars in all images = 100 μm

**FIGURE 7:**
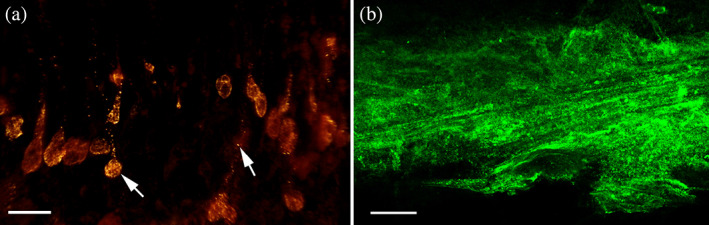
*Arion vulgaris*, lectin affinity tests. (a) The secretory material of one or more gland types of the ventral area shows a strong affinity for α‐L‐fucose (lectin UEA II) (white arrows), while the remaining epithelium lacks a clear reaction to this sugar moiety. (b) Also, the isolated ventral mucus reacts positively to α‐L‐fucose (lectin UEA II) only. Scale bar in a and b = 50 μm

Unlike the lateral glands, one of the smaller ventral gland types (representing either the A1v, A2v or A3v types based on its size) shows positive PAS staining, indicating the presence of hexose‐containing mucosubstances (Figure 6a) and polyanionic molecules (Safranin O staining; Figure 6b). A more detailed sugar characterization by lectins indicates a strong binding affinity for fucose (only UEA II but not UEA I) (Table 1) in the gland content and the duct (Figure 7a) as well as in the secreted mucus (Figure 7b) of any of the three small gland types. Beside UEA II, also a small affinity to STL (specific for N‐acetylglucosamine) (Table 1) could be confirmed in the gland region. All other applied lectins showed no clear affinity with the gland or its secretion.

Besides hexose‐containing the secretory content also reacts to sulphated and carboxylated acidic mucosubstance stainings: To Alcian blue at pH 1.0 (data not shown) and pH 2.5 (Figure 6c) as well a metachromasia to Toluidine blue at pH 4.3 (data not shown), indicating the presence of acidic mucosubstances in one or more ventral glands.

The largest ventral gland type (A4v) shows a positive reaction to calcium only with Alizarin red S, but not by the von Kossa method (Figure 6d), while all other applied histochemical stainings show no reactivity. Some calcium deposits can be found in the ventral subepidermal muscle layer. The secretory content of the ventral glands shows no reactivity to Biebrich scarlet, Arnow reaction, and von Kossa staining, but the isolated ventral mucus shows a strong affinity to calcium (Alizarin red S and von Kossa method) and lipid (Sudan black B; Figure 6e).

### 3.6 EDX‐analyses of the ventral mucus

The element concentration is similar to that of the lateral side, whereby carbon (19–47 at.%), nitrogen (3–14 at.%), and oxygen (8–40 at.%) are predominantly present (Table 2). Values can be measured for chlorine (0.3 at.%, locally crystals contain values above 2–11 at.%) and potassium (1 at.%, locally crystals contain values above 4–14 at.%) (Figure S1d–f), while for sodium, magnesium, phosphorus, sulphur, and calcium again low concentrations could be measured in the mucus.

## 4 DISCUSSION

During the last decades, much attention has been paid to the gastropod species *Arion vulgaris* as this species is considered the worst pest species by private garden owners and commercial agriculturists. Its highly viscous ventral mucus surely contributes to their geographical dispersal (Gural‐Sverlova & Gural, 2011) as its opportunity to overcome many types of obstacles, including sharp surfaces and super anti‐adhesive surfaces (Shirtcliffe et al., 2012).

### 4.1 Lateral subepithelial gland system

Published data varies concerning the number of epithelial mucus gland types, not only in *arionid species but* Arionoidea in general. Moreover, the lateral gland types are frequently disregarded as they do not contribute to its locomotion or adhesion, nor to shell building, as has been described for the dorsal glands of helicid species (Campion, 1961; Greistorfer et al., 2017). Summarising the data from the literature (Table 3), the current study confirms five gland types located laterally in *Arion vulgaris*, although in other arionid species only four (*Limax ecarinatus*, Cook & Shirbhate, 1983), three (*Limax maximus*, Herfs, 1921) or two gland types (*Arion ater*, Barr, 1927; Wondrak, 1967; *Lehmania poirieri*, Arcadi, 1967; *Meghimatium fruhstorferi*, Yamaguchi et al., 2000) have been observed. Using histochemical methods, exclusively acidic molecules were found in the present investigation into *Arion vulgaris*, while in other arionoidea, additionally acidic glycoproteins and/or sugars are *synthesised. In lateral glands of Arion vulgaris*, positive reactions for calcium (Alizarin red S and the von Kossa method) could be confirmed, a result that has not been available for other arionids.

**TABLE 3:** Overview of the epithelial glands in different arionoidea species

	*Arion vulgaris*	*Arion ater* (syn. *Arion empiricorum*)	*Arion rufus & Arion vulgaris*	*Arion rufus*		*Limax ecarinatus* (syn. *Limax pseudoflavus*)	*Limax maximus*	*Lehmania poirieri*	*Meghimatium fruhstorferi*
	(present study)	Barr (1927)	Wondrak (2012)	Wondrak (1967)	Chétail and Binot (1967)	Cook and Shirbhate (1983)	Herfs (1921)	Arcadi (1967)	Yamaguchi, Seo, and Furuta (2000)
Lateral epithelium	5	2	n.d.	1	n.d.	3	3	2	2
	**A1l**	**Calcic**		**Mantle gland**		**Goblet‐shaped**	**Calcic**	**Basket cell complex**	**Round mucous**
	Positive to AB pH 1.0, AB pH 2.5 and TB pH 4.0	Lime mucus		n.d.		Positive to PAS/AB pH 2.5, Alcian blue (pH 0.5/eosin), Alcian blue (pH 0.5)/Alcian yellow (pH 2.5))	Lime mucus, keep skin moist	Positive to PAS	Positive to lectin WGA and SBA
	**A2l**	**Unicellular mucous**				**Clavate‐shaped**	**Mucous**	**Granular cell complex**	**Tubular**
	Positive to AB pH 1.0, AB pH 2.5 and TB pH 4.0	Mucus in general (weak acid fuchsin)				Positive to PAS, Sudan B	Not stained, used for locomotion	Positive to PAS, TB pH 4.0	Positive to AB pH 2.5, lectin RCA
	**A3l**					**Spatulate‐shaped**	**Acidophile**		**#**
	‐					Positive to PAS, Sudan B	Defence secretion		
	**A4l**								
	Positive to AB pH 2.5 and TB pH 4.0								
	**A5l**								
	Positive to AB pH 1.0, AB pH 2.5 and TBpH 4.0								
Peripodial groove	1	1	1	n.d.	n.d.	1	n.d.	n.d.	n.d.
	**At**	**Peripodal**	**Anterior margin of mantle**			**Oval‐shaped**			
	n.d.	n.d.	Alcian blue pH 4.0			Positive to PAS			
Ventral epithelium	4	1	2	2	5	5	1	1	2
	**A1v**	**Unicellular mucous**	**Pedal protein gland**	**Sole gland**	**Cell type I**	**Round‐shaped (locality: Sole, groove)**	**Mucous**	**Granular cell complex**	**Round mucous**
	Positive to PAS/AB pH 1,0 and 2.5, TB pH 4.0	Mucus in general (weak acid fuchsin)	Weak to PAS, lectin PNA, RCA, HPA	n.d.	Positive to PAS, TB pH 4.5, Sudan B	Positive to PAS/AB pH 2.5	Not stained, used for locomotion	Positive to PAS, TB pH 4.0	Lectin WGA and SBA
	**A2v**	**Pigmentary**	**Ventral head surface**	**Lateral gand**	**Cell type II**	**Oval‐shaped (sole, head, flank)**			**Tubular**
	Positive to PAS/AB pH 1,0 and 2.5, TB pH 4.0	Black pigments	Weak to PAS, lectin RCA, HPA	n.d.	Positive to PAS, TB pH 4.5	Positive to PAS/AB pH 2.5			Positive to AB pH 2.5, lectin RCA)
	**A3v**				**Cell type IIIa**	**Oval‐shaped (median foot)**			
	Positive to PAS/AB pH 1,0 and 2.5, TB pH 4.0				Positive to PAS, Sudan B	Positive to AB pH 2.5			
	**A4v**				**Cell type IIIb**	**Polygonal‐shaped (inferior foot)**			
	Positive to PAS/AB pH 1,0 and 2.5, TB pH 4.0				Positive to PAS, Sudan B	Positive to PAS/AB pH 2.5			
					**Cell type IV**	**Oval‐shaped (superior foot)**			
					Mucoproteins	Positive to PAS			

*Note:* The number in the first line list the number of glands found in the respective epithelial layer, subsequently its gland nomenclature according to the authors.

Abbreviations: AB, alcian blue G8X; n.d., not determined; PAS, periodic acid schiff; TB, toluidine blue O.

### 4.2 Transitional subepithelial glands

Gland type At, found in the transitory zone of *Arion* in the present study, almost certainly corresponds to the peripodial gland of *A. ater* (Chétail & Binot, 1967), *A. vulgaris*, and *A. rufus* (Wondrak, 2012) and the oval‐shaped gland type of *Limax ecarinatus* (Cook & Shirbhate, 1983). No information about such a gland type or its chemical content has been presented for the other species nor its involvement in mucus formation been confirmed.

### 4.3 Ventral subepithelial gland system

While in helicid species only two gland types are classified ventrally (Table 2), in some arionid species up to five different gland types have been documented (Table*3* *)*. In contrast, *Limax maximus and L. poirieri* contain only one gland type in the sole epithelium (Arcadi, 1967; Herfs, 1921 *)*. As in the case of the lateral gland types, the ventral glands also differ with regard to their secretory content. In most species, at least one ventral gland can be detected that contains acidic mucosubstances (positive PAS and Alcian blue or Toluidine blue staining, reactivity to lectins; Table 1), while no data are given for *A. ater* or *L. maximus*. Furthermore, most species additionally contain a gland type synthesising hexose‐containing mucosubstances (positive PAS staining, presence of lectins such as RCA, WGA, HPA, etc.), which is lacking in the species investigated here. The affinity to UEA II, PNA, WGA, VVL and GNA confirms the presence of sugars in the ventral mucus of *Arion vulgaris*. This indicates that PAS staining is not sensitive enough to detect the presence of small sugar amounts.

Some lectins (PNA, WGA) did not show an affinity to the lateral mucus but a reactivity in the glands. It could not be excluded that the lectin amount in the mucus was lower than in the gland cells.

In arionid species, it is proposed that the ventral (trail) mucus for locomotion is not only produced by the ventral gland types, as been supposed for the helicid species (Greistorfer et al., 2017; von Byern et al., 2018). Additionally, the suprapedal gland system, located within the ventral body cavity, contributes to the secretion of trail mucus (Barr, 1927; Wondrak, 2012). The system contains only one gland type, which stains positive for sulphated and carboxylated acidic mucosubstances and specific sugars moieties such as galactose (lectin GSL‐1 B4 and lectin *RCA*) and N‐acetyl‐α‐D‐galactosaminyl (lectin *Helix pomatia*), but is negative for PAS staining (Wondrak, 2012). In our study, we did not re‐examine the suprapedal gland, but focused on the weakly characterised subepithelial gland system.

Beside similarities to other *arionid species*, the *Arion vulgaris* specimens also share similarities to the gland morphologies of helicid species like *Helix pomatia* and *Cepaea hortensis* (Table 2). All three possess a lateral/dorsal gland type with ice floe‐like granules (A1l, C1d, H1d), whereas in *Arion* this gland additionally occurs ventrally (A1v). Besides the differences in the gland number (*Arion* = 5 lateral, 1 transition, 4 ventral; *Helix* = 3–4 dorsal, 2 ventral; Greistorfer et al., 2017; *Cepaea* = 4 dorsal, 2 ventral, von Byern et al., 2018), their subepidermal glands also vary chemically:

The ventral gland types of *Arion* produce exclusively acidic glycoproteins, as does *Helix* (Greistorfer et al., 2017), with an affinity for the lectins UEA II, WGA, VVL and GNA. In *Cepaea*, one gland type shows reactivity to acidic proteins only (von Byern et al., 2018), and also in *Cepaea* (as well as in *Helix*), the lectin GNA shows affinity to the ventral mucus. Dorsally, the helicid gland types likewise react to acidic mucosubstances (*Helix* and *Cepaea*) or basic proteins (*Cepaea* only; Greistorfer et al., 2017; von Byern et al., 2018), likely serving as hydrogel‐like lubricants that reduce the friction force between the soft skin and the hard shell (Herfs, 1921; Werneke, Swann, Farquharson, Hamilton, & Smith, 2007). In *Arion*, only acidic mucosubstances could be detected laterally, suggesting a defence system against predators or bacteria, as discussed earlier (Barnhart, 1983; Pawlicki et al., 2004).

In a detailed report, Campion (1961) described how helicid snails produce and secrete calcium to build their shell and epiphragm. Distinct calcium depots can be observed among the dorsal glands in *Helix* and *Cepaea* (Greistorfer et al., 2017; von Byern et al., 2018). On the other hand, calcium and metals like iron or copper or other elements (e.g., potassium, sulphur, phosphorus) could also be detected in the mucus of terrestrial gastropods such as *Arion subfuscus* (Braun, Menges, Opoku, & Smith, 2013) and marine species such as *Lottia limatula* (Smith, Quick, & St.Peter, 1999). These elements are not only involved in shell formation, but also play a role in the cross‐linkage and viscosity of the mucus (Pawlicki et al., 2004; Werneke et al., 2007), as described for the *Mytilus* byssal system (Waite, Holten‐Andersen, Jewhurst, & Sun, 2005). More precise analyses with ICP‐MS are necessary to study the presence of heavy metals (iron, copper, manganese) in the presently investigated *Arion* mucus, as measured for *A. subfuscus* (Braun et al., 2013), since the performed EDX measurement may not be sufficiently sensitive.

Currently, the high levels of potassium and chlorine found locally in the ventral and lateral mucus in *Arion vulgaris* (present study), as well as in the two helicid species examined earlier (Greistorfer et al., 2017; von Byern et al., 2018), cannot be explained. Analyses of the dorsal mucus of *H. pomatia* indicate that the high concentrations of potassium and magnesium are not exclusively components of the haemolymph, but are also released by the epithelial gland cells (Burton, 1965). However, there are no suggestions about the chlorine origin as well as the function of potassium and chlorine in the synthesis of the mucus. Further studies with differently reared and fed animals are necessary to exclude a nutrient or cultivation effect.

## Supporting information


**Figure S1** **Elemental analysis of the lateral and ventral *Arion* secretory material**. **A)** SEM analyses indicate the presence of crystals in the lateral mucus, which **B)** appears to consist of chloride. **C)** Also, the element potassium is uniformly distributed in the lateral mucus (arrow). **D)** In the ventral mucus like‐wise crystals could be observed. **E)** The element chloride is not uniformly distributed but appears patched. **F)** The element potassium is present in high abundance (arrow). Scale bars in A and D = 50 μm, in B, C, E and F = 20 μm.

## Data Availability

My article type does not require one
