# Supplementary figures and images for: Characterization of the Arion vulgaris pedal gland system
Source: J Morphol. 2020 Jul 10;281(9):1059–71. doi: 10.1002/jmor.21231 (PMC7496283; doi:10.1002/jmor.21231)

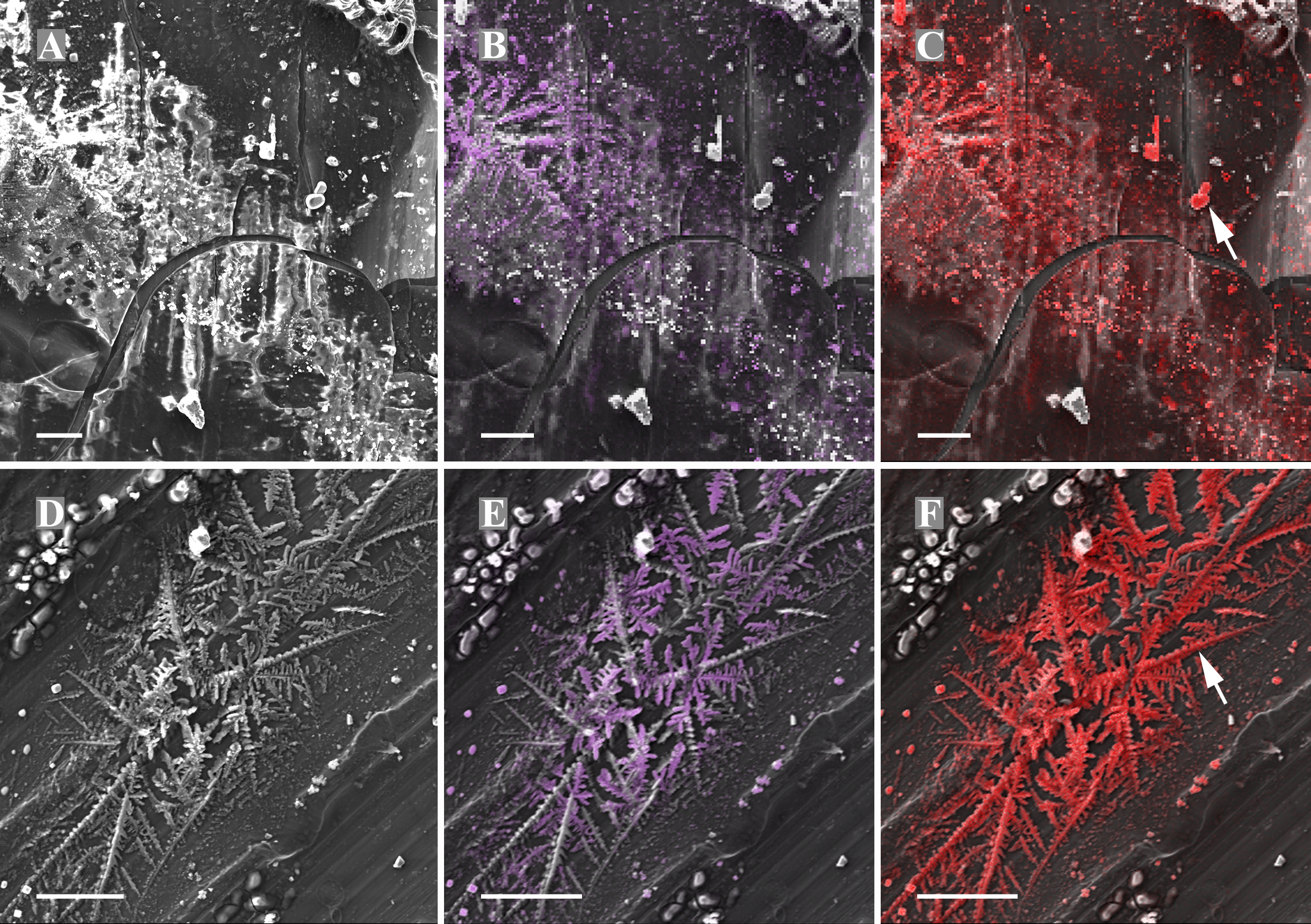

Supplement: Supplementary file 1 — Figure S1 Elemental analysis of the lateral and ventral Arion secretory material. A) SEM analyses indicate the presence of crystals in the lateral mucus, which B) appears to consist of chloride. C) Also, the element potassium is uniformly distributed in the lateral mucus (arrow). D) In the ventral mucus like‐wise crystals could be observed. E) The element chloride is not uniformly distributed but appears patched. F) The element potassium is present in high abundance (arrow). Scale bars in A and D = 50 μm, in B, C, E and F = 20 μm. [file JMOR-281-1059-s001.tif]
